# Foodborne intestinal protozoan infection and associated factors among patients with watery diarrhea in Northern Ethiopia; a cross-sectional study

**DOI:** 10.1186/s41043-018-0137-1

**Published:** 2018-03-02

**Authors:** Birhane Berhe, Gessessew Bugssa, Sena Bayisa, Megbaru Alemu

**Affiliations:** 1 Department of Bio-Medical Laboratory Science, College of Health Science, Adigrat University, Adigrat, Ethiopia; 2 Department of Medical Parasitology and Entomology, Institute of Biomedical Sciences, College of Health Science, Mekelle University, Mekelle, Ethiopia; 3 Department of Medical Microbiology, Immunology and Parasitology, Bahir Dar University, Mekelle, Ethiopia; 4 Department of Microbiology, Immunology and Parasitology, Bahir Dar University, PO Box 79, Bahir Dar, Ethiopia

**Keywords:** Food borne, Protozoa, Diarrheal, Ethiopia

## Abstract

**Background:**

Intestinal protozoa are parasites transmitted by consumption of contaminated water and food and mainly affect children and elder people and cause considerable health problems. They are the leading causes of outpatient morbidity due to diarrhea in the developing countries. So, assessing water and food source of diarrheal patients and identifying the main associated factors for transmission of protozoan parasitic infections help for effective control measures of protozoan infections. Hence, the current study was aimed at determining the prevalence of foodborne intestinal protozoa infections and associated factors among diarrheic patients in North Ethiopia.

**Methods:**

A health facility based cross-sectional study was conducted among 223 patients with watery diarrhea in four selected government health facilities in North Ethiopia from November 2016–June 2017. A structured questionnaire was used to collect data on socio-demography of study participants and factors associated with foodborne protozoa infections. The diarrheic stool samples were collected, transported, and processed using direct wet mount, formal-ether concentration and modified ZiehlNeelson staining methods. The data were analyzed using SPSS version 21 and descriptive statistics, bi-variate, and multivariate logistic regressions were computed. *P*-value < 0.05 at 95% confidence interval was considered statistically significant.

**Results:**

The overall prevalence of foodborne protozoa infection was 101 (45.3%). The predominant protozoa species identified was *Entamoeba histolytica/dispar* 55 (24.7%), followed by *Giardia intestinalis* 25 (11.2%) and *Cryptosporidium* species 5 (2.2%). The highest proportion of protozoa infection was observed among males (23.3%) and the age group 15–24 years (13.5%). Statistically significant associations were observed between foodborne protozoan infection and not using any type of recipe to decontaminate salads and fruits (AOR = 2.64, 95 CI: 1.34–5.19, *P* = 0.005) and using vinegar as a decontaminant (AOR = 2.83, 95 CI: 1.24–6.48, *P* = 0.014). Eating out (meals at a restaurant) on the other hand was found to be protective for foodborne protozoan infection (AOR = 0.43, 95 CI: 0.23–0.78, *P* = 0.006).

**Conclusion:**

Our study revealed that foodborne protozoa infections are of public health significance in the study area. Vinegar, which is frequently used as a recipe for decontaminating salads and fruits, is inversely related to foodborne protozoa parasite infection .

## Background

Intestinal protozoa are a diverse group of unicellular organisms inhabiting the intestinal tract of humans [1]. Infections usually occur through ingestion of cysts/oocysts contaminating food or drinking water [2, 3]. Infection with intestinal protozoa parasites have worldwide distribution and particularly common in tropics and sub-tropics areas of the world with millions of cases of diarrhea occurring in each year. There are a number of intestinal protozoa that cause diarrhea, but *Entamoeba histolytica*, *Giardia intestinalis* and *Cryptosporidium* species are the most important causes of diarrhea [4–6].

Giardiasis is the most frequently reported intestinal protozoan disease in the world, with about 280 million symptomatic cases and 2.5 million annual deaths each year [7]. An estimated 748,000 cryptosporidiosis cases also occur annually, though less than 2% are reported due to poor sensitivity of the routine direct wet mount microscopy. Hospitalizations resulting from cryptosporidiosis cost an estimated 45.8 million dollars per year [8]. Amoebiasis is also a common cause of intestinal protozoan infections throughout the tropics.

In Ethiopia, morbidity and mortality due to diarrheal diseases remain to be the main public health problem that needs attention in the country. The lack of safe drinking water, poor environmental sanitation, and poor socioeconomic status are responsible for more than 800 million cases and 4.5 million deaths associated with diarrheal diseases occurring every year [9, 10]. Malnutrition, anemia, growth restrictions, cognitive delays, irritability and increased susceptibility to other infections are some of the consequence of diarrhea morbidities [11, 12].

Several studies have addressed the epidemiology of intestinal parasites mainly in apparently healthy school children in Ethiopia [13–19], yet there exists paucity of data on the diarrheal causes of food borne intestinal protozoan parasites in the country in general and North Ethiopia in particular. In addition, the pattern of intestinal parasitism in a population with diverse groups of people was not illustrated. Furthermore, most previous studies have not employed the modified Ziehl Neelson staining method for the diagnosis of opportunistic intestinal coccidian parasites and consequently, they were under-reported so far. Hence, we planned to determine the magnitude of food borne protozoa species associated with diarrhea among patients with watery diarrhea in North Ethiopia.

## Methods

### Study design, period and area

This cross-sectional study was conducted in selected government health facilities in Mekelle City from November 2016 to June 2017. Mekelle city is located 780 km north of Addis Ababa at a latitude and longitude of 13°29′N39°28′E with an elevation of 2084 m above sea level. The average annual temperature is 18.0 °C. Based on the 2007 census conducted by the Central Statistical Agency of Ethiopia (CSA), the city has a total population of 215,914 (104,925 men and 110,989 women). The main source of water is ground water from 19 boreholes that range from 32 to 250 m deep. There were 13 governmental health facilities (2 hospitals and 11 health centers) in the city [20].

### Study participants

The study participants for this particular research work were volunteer diarrheic outpatients who were requested for stool examination in the health facilities during the study period. The patients visited the health facilities with a chief complaint of diarrhea. Those patients who took medication for intestinal parasites within 1 month prior to the study were excluded. Moreover, patients who were severely ill and were unable to give stool sample were excluded from the study. The study participants were selected by a non-probability convenient sampling.

### Study size

A sample size of 226 was determined using single population proportion statistical formula by the following assumptions: 95% level of confidence, 5% margin of error and P (proportion) of 0.16 [21], and non-response rate of 10%. From a total of 13 health institutions of Mekelle City, one hospital and three health centers were selected randomly. Accordingly, Mekelle hospital, Mekelle health center, Semien health center and Adishimdihun health center were included in this study. The 226 study subjects were proportionally allocated to the health facilities based on the size of patient flow within 3 months prior to our study.

### Data collection

#### Questionnaire

Data on demographic characteristics of study participants and associated factors of protozoan infections were collected using structured and pre-tested questionnaire. The questionnaire was developed based on previous research works and pre-tested in Ayder referral hospital. The face-to-face interview was conducted by trained data collectors.

#### Stool examination

Labeled stool containers with tight covers bearing serial numbers of the subjects were supplied for the study participants. Fecal specimens were processed by wet mount, Formol-ether concentration (FEC) and modified Ziehl Neelson staining methods.

#### Wet mount technique

Fresh stool samples (approximately 2 mg of stool) were put on a slide with the wooden applicator and emulsified with a drop of physiological saline (0.85%). The preparation was then covered with a cover slide and examined at 10× and 40× microscopic objectives [6].

#### Formol ether concentration technique

Approximately 0.5 g of feces was mixed with 10 ml of normal saline and the mixed stool was strained via gauze into a funnel. The strained contents were collected in a centrifuge tube. About 2.5 ml of 10% formaldehyde and 1 ml of ether were then added and centrifuged at 1000 g for 3 min. The supernatant was removed and a drop of the sediment was covered with cover glass for a microscopic investigation [6].

#### Modified Ziehl-Neelsen technique

Thin fecal smears were prepared from fresh fecal specimens and air dried. Following fixation with methanol for 5 min, the primary stain (carbon fuchsin) was applied for 10 min. The smear was washed and decolorized with 1% acid alcohol. The counter stain (0.5% malachite green) was then applied for 30 min. The smear was washed off, dried and examined accordingly [6].

### Quality control

The Questionnaire was pre-tested before actual data collection. Diarrheic specimens were processed within 15 min of collection to demonstrate the motile protozoa trophozoites. The collected data were checked for consistency and accuracy on a daily basis. All the laboratory procedures were conducted as per the Standard Operating Procedures (SOPs).

### Statistical analysis

Data were entered and analyzed using statistical software package (IBM Comp. released2011. IBM SPSS statistics for windows, version 20 Armonk, NY: IBM comp). Data were summarized using descriptive statistics. Bi-variate and multi-variate regression tests were employed to measure the strength of association between dependent and independent variables. Variables with *P* < 0.2 in the bi-variate logistic regression were transferred to multi-variate regression analysis and the AOR was computed to control potential confounders.. *P*-value less than 0.05 was considered statistical significant.

## Results

### Demographic characteristics

A total of 223 diarrheic outpatients were enrolled with a response rate of 98.7%. Out of the total respondents, 124 (55.6%) were males. The median age of study participants was 21 years, ranging 1–80 years (Table 1). Majority of the study participants were in the age group of 15–24 years (24.7%), high school graduates (33.6%) and unemployed (71.3%).

**Table 1:** Demographic characteristics of the study participants in selected government health facilities of Mekelle City, North Ethiopia, 2017

Variables	Frequency	Percent (%)
Sex		
Male	124	55.6
Female	99	44.4
Age (Yrs)		
< 5	36	16.1
5–14	46	20.6
15–24	55	24.7
25–34	44	19.7
35–44	16	7.2
> =45	26	11.7
Occupation		
Civil servant	23	10.3
Self-employed	37	16.6
Farmer	4	1.8
Unemployed	159	71.3
Educational level		
Illiterate	38	17
Read and write	47	21.1
Primary school	34	15.2
High school	75	33.6
College and above	29	13

### Prevalence of food borne intestinal protozoa

The overall prevalence of intestinal protozoa infection was 45.3% (101/223) and higher infection rates were recorded for males (23.3%) and the age group 15–24 years (13.5%) (Table 2). *E. histolytica/dispar,* 55 (24.7%) was the predominant parasite followed by *G. intestinalis*, 25 (11.2%) and *Cryptosporidium* species, 5 (2.2%). The rate of double infection accounted for 6.7% (15/223), the predominant co-existent infection being *E. histolytica/dispar* and *G. intestinalis* (4.9%) (Table 2).

**Table 2:** Distribution of food borne protozoa by sex and age among diarrheic patients in selected government health facilities of Mekelle City, NorthEthiopia, 2017

Parasite species	Gender		Age (Yrs)					
	Male n (%)	Female n (%)	< 5 n (%)	5–14 n (%)	15–24 n (%)	25–34 n (%)	35–44 n (%)	≥ 45 n (%)
Single infection								
*G. intestinalis*	15 (6.7)	10 (4.5)	5 (2.2)	6 (2.7)	8 (3.6)	3 (1.3)	1 (0.4)	2 (0.9)
*E. histolytica*	28 (12.5)	27 (12.2)	10 (4.5)	12 (5.4)	15 (6.7)	8 (3.6)	5 (2.2)	5 (2.2)
*Cryptosporidium* spp	4 (1.8)	1 (0.4)	–	2 (0.9)	2 (0.9)	1 (0.4)	–	–
Double infection								
*G. intestinalis + E. histolytica*	5 (2.2)	6 (2.7)	–	1 (0.4)	4 (1.8)	1 (0.4)	1 (0.4)	4 (1.8)
*G. intestinalis* + *Cryptosporidium* spp	1 (0.4)	–	–	–	–	1 (0.4)	–	–
*E. histolytica Cryptosporidium* spp	1 (0.4)	2 (0.9)	–	–	1 (0.4)	1 (0.4)	–	–

The prevalence of *G. intestinalis* and *Cryptosporidium* species among males was 6.7% and 1.8%, respectively. Higher prevalence of *G. intestinalis and E. histolytica/dispar* was found in the age group 15–24 years, 3.6% and 6.7%, respectively (Table 2).

### Factors associated with food borne protozoan infections

The prevalence of food borne protozoa infection was higher among those who had no regular hand washing habit before a meal, those whose house hold water was sourced from rivers and those whose toilets were < 5 m from their kitchens. However, the difference was not statistically significant (*P* > 0.05). All variables with *P* < 0.2 in the bi-variate logistic analysis were taken to multiple logistic regressions. In multivariate analysis, using vinegar as a decontaminant for vegetables and fruits (AOR = 2.83, 95% CI: 1.24–6.48, *P* = 0.014) and not using any type of recipe as a decontaminant (AOR = 2.64, 95 CI: 1.34–5.19, *P* = 0.005) were significant indicators of intestinal protozoan infection. ‘Eating out’ (eating at a restaurant) was significantly associated with a lower odds of intestinal protozoa infection (AOR = 0.43, 95 CI: 0.23–0.78, *P* = 0.006) (Table 3).

**Table 3:** Bi-variate and multi-variate logistic regression analysis of factors associated with foodborne protozoan infection among diarrheic patients in selected government health facilities of Mekelle City, North Ethiopia, 2017

Variables	Parasite status		COR (95% CI)	*P*-value	AOR (95%CI)	*P*-value
	Positive N (%)	Negative N (%)				
Water treatment						
No treatment	39 (45.9)	46 (54.1)	0.9 (0.5–1.6)	0.68		
Boiling/filtration	14 (35)	26 (65)	0.6 (0.26–1.2)	0.14	0.8 (0.34–1.8)	0.8
Chemical treatment	48 (49)	50 (51)	1		1	
Water source for drinking and cooking						
Protected water	95 (45.2)	115 (54.8)	1			
Unprotected water	6 (46.2)	7 (53.8)	1.04 (0.34–3.12)	0.95		
Regular hand washing						
Yes	99 (45)	121 (55)	1			
No	2 (66.7)	1 (33.3)	2.44 (0.41–4.6)	0.47		
Feces disposal/defecation						
Open defecation	13 (56.5)	10 (43.5)	2.1 (0.85–5.03)	0.11	0.42 (0.16–1.22	
Pit latrine	32 (58.2)	23 (41.8)	2.2 (1.18–4.16)	0.014	0.95 (0.32–2.8)	0.92
Flash latrine	56 (38.6)	89 (61.4)	1		1	
Distance of kitchen from toilet (meter)						
20–30	17 (42.5)	23 (57.5)	1			
10–20	35 (45.5)	42 (54.5)	1.13 (0.52–2.44)	0.76		
5–10	32 (42.7)	43 (57.3)	1.01 (0.46–2.19)	0.97		
< 5	17 (54.8)	14 (45.2)	1.62 (0.64–4.23)	0.3		
Decontaminant used for vegetables and fruits						
Lemon	17 (28.8)	42 (71.2)	1		1	
Vinegar	24 (54.5)	20 (45.5)	3 (1.31–6.72)	0.009	2.83 (1.24–6.48)	0.014
None	60 (50)	60 (50)	2.5 (1.28–4.82)	0.008	2.64 (1.34–5.19)	0.005
Eating at restaurant						
Yes	45 (35.2)	83 (64.8)	0.38 (0.22–0.65)	0.000	0.43 (0.23–0.78)	0.006
No	56 (58.9)	39 (41.1)	1		1	

*COR* crude odds ratio, *CI* confidence interval, *AOR* Adjusted odds ratio, 1(referent)

## Discussion

Intestinal protozoa are considered important emerging pathogens contributing to diarrheal disease outbreaks in developing nations where declining water quality is a persistent problem [22]. In our study, the overall prevalence of protozoan infection was 45.3%. Our finding was higher than the study conducted in Southern Ethiopia 16.6% [10], Southwestern Iran 32.2% [23] and Western Iran 37.5% [24]. However, it was lower than findings from South Africa 68% [25] and Lebanon 85% [26] among diarrheic patients. These inconsistencies might be due to differences in living standards of study participants, environmental sanitation, hand washing and hygiene practices of the populations and usage of latrine and waste disposal [10, 23]. The stool examination techniques utilized in different research works might also have contributed for such inconsistencies [23, 27–29].

*E. histolytica/dispar* with a prevalence of 24.7% was the predominant species among diarrheic patients in our study. This was higher than studies in Ethiopia; Jimma 5.6% [15], Gondar 10.3% [30], and elsewhere; Saudi Arabia 4.7% [31], Malaysia 0.4% [32], Italy 4.1% [33] and Myanmar 6.2% [34]. The higher prevalence of *E. histolytica/dispar* in this study might be due to poor access to safe drinking water supplies which is testified by the fact that about half of the study participants used unprotected water for drinking and cooking in our study. Giardiasis accounted for 11.2% in our study which was higher than reports from Philippines 2% [35], India 5% [36] and China 6.8% [37]. But it was lower than findings from Ethiopia 25.5% [38], and South Africa 20.8% [25].

In this study, the prevalence of *Cryptosporidium* species (2.2%) was lower than reports from India 16.3% [36] and China 13.49% [37]. The observed difference could be due to absence of advanced molecular technique to detect *Cryptosporidium* species. The prevalence of *Giardia intestinalis* and *Cryptosporidium* species was higher in males than females. This was consistent with the study in Lebanon [26], Iraq [39], Nigeria [40], and Saudi Arabia [41]. This might be due to the fact that males have more exposure as they are frequently engaged in practices such as dealing with farm animals as zoonotic transmission could happen.

In the multi-variate analysis, study participants who did not use any type of recipe to decontaminate vegetables and fruits were 2.6 times at higher risk of acquiring foodborne intestinal protozoa infection. This might be due to the practice of growing of vegetables and fruits in gardens where night soils and untreated human feces is used as fertilizer as testified by our finding that more than 10% of the participants reported open defecation. In addition, long survival of protozoa cysts/oocysts in moist soils might be attributed to risk of infections. Similarly, the risk of protozoan infection was 2.8 times higher among those who used vinegar compared to those who use lemon as a recipe to clean fruits and vegetables. This might be an indication that the routinely used vinegar to decontaminate fruits and vegetables has poor killing effect to cysts of foodborne protozoa parasites. Reduced cysticidal activity of vinegar was documented at low temperatures in previous studies [42, 43]. In addition, the giardiacidal activity of lemon juice was indicated in a previous study [43].

Our study revealed that ‘eating out’ (eating in a restaurant regularly) was found to be protective for protozoa infection. For instance, the odds of acquiring protozoan infection was 0.43 times higher among those who regularly eat at a restaurant than those who cook and consume at their homes. It is supported by findings elsewhere that home is the location associated with significant foodborne illness risk. Home is the place to prepare most of the food we consume, thereby increasing the possibility of food mishandling [44]. Similarly, people in groups are known to be at risk of foodborne infections. For instance the immunocompromised, young infants and pregnant women live together thereby exacerbating the transmission of foodborne pathogens [45]. Furthermore, most consumers do not perceive themselves or someone in their families to be susceptible to foodborne illness [46], or do not follow all recommended food safety practices [47], and consequently they do not take sufficient precautions.

The present study also showed that the magnitude of protozoan infection was higher among participants whose water for drinking and cooking was collected from unprotected sources such as rivers. This was in agreement with the report in Cote d’Ivoire [48]. This might be an indication for the incomplete separation of human and animal waste with water sources that are used for drinking in the area [49]. In addition, the resistant cysts of the parasites for routine chlorination might have contributed for the relatively higher infection rates among those who used protected water sources as well.

## Conclusion

Foodborne intestinal protozoa are of public health concern in the study area. Using vinegar as a decontaminant to eat fruits and vegetables was not protective of protozoa infections. Eating at a restaurant in a regular basis on the other hand was associated with lower odds of infection of foodborne protozoa parasites. Heath education should be given on handling of food at home and safe potable water should be provided to the society to interrupt the transmission of foodborne protozoan parasitic infections. Household latrines should also be built far from kitchens.

### Limitation of the study

We were unable to identify the species of *Cryptosporidium* due to lack of molecular techniques. Other possible causes of diarrhea such as bacteria and/or viruses were not also illustrated.

## Abbreviations

CI: Confidence interval
CSA: Central Statistical Agency
OR: Odds ratio
SPSS: Statistical Product and Service Solutions
