# Correction to: Foodborne intestinal protozoan infection and associated factors among patients with watery diarrhea in Northern Ethiopia; a cross-sectional study

**DOI:** 10.1186/s41043-019-0176-2

**Published:** 2019-08-05

**Authors:** Brhane Berhe, Gessessew Bugssa, Sena Bayisa, Megbaru Alemu

**Affiliations:** 1 Department of Bio-Medical Laboratory Science, College of Health Science, Adigrat University, Adigrat, Ethiopia; 2 Department of Medical Parasitology and Entomology, Institute of Biomedical Sciences, College of Health Science Mekelle University, Mekelle, Ethiopia; 3 Department of Microbiology, Immunology and Parasitology, Bahir Dar University, PO Box 79, Bahir Dar, Ethiopia

**Correction to: J Health Popul Nutr (2018) 37: 5**

**https://doi.org/10.1186/s41043-018-0137-1**

Unfortunately, the original version of this article [1] contained errors. The first author name was included incorrectly. The correct spelling, Brhane Berhe, has been included in the author list above. Additionally, the corresponding author was incorrectly affiliated. The correct affiliation has been included in the author details below.

These errors have now been updated in this correction.
